# Impairments in cognitive performance in chronic fatigue syndrome are common, not related to co-morbid depression but do associate with autonomic dysfunction

**DOI:** 10.1371/journal.pone.0210394

**Published:** 2019-02-05

**Authors:** Lucy J. Robinson, Peter Gallagher, Stuart Watson, Ruth Pearce, Andreas Finkelmeyer, Laura Maclachlan, Julia L. Newton

**Affiliations:** 1 School of Psychology, Newcastle University, Newcastle, United Kingdom, and Northumbria Healthcare NHS Foundation Trust, Newcastle, United Kingdom; 2 Institute of Neuroscience, Newcastle University, The Henry Wellcome Building, Framlington Place, Newcastle upon Tyne, United Kingdom; 3 Academic Psychiatry and Regional Affective Disorders Service Newcastle University, Newcastle upon Tyne, United Kingdom, and Northumberland, Tyne and Wear Foundation Trust, Wolfson Research Centre, Campus for Ageing and Vitality, Newcastle upon Tyne, United Kingdom; 4 Institute of Cellular Medicine, Newcastle University, Newcastle, United Kingdom; 5 Newcastle Hospitals NHS Foundation Trust, Newcastle, United Kingdom; Texas Technical University Health Sciences Center, UNITED STATES

## Abstract

**Objectives:**

To explore cognitive performance in chronic fatigue syndrome (CFS) examining two cohorts. To establish findings associated with CFS and those related to co-morbid depression or autonomic dysfunction.

**Methods:**

Identification and recruitment of participants was identical in both phases, all CFS patients fulfilled Fukuda criteria. In Phase 1 (n = 48) we explored cognitive function in a heterogeneous cohort of CFS patients, investigating links with depressive symptoms (HADS). In phase 2 (n = 51 CFS & n = 20 controls) participants with co-morbid major depression were excluded (SCID). Furthermore, we investigated relationships between cognitive performance and heart rate variability (HRV).

**Results:**

Cognitive performance in unselected CFS patients is in average range on most measures. However, 0–23% of the CFS sample fell below the 5th percentile. Negative correlations occurred between depressive symptoms (HAD-S) with Digit-Symbol-Coding (r = -.507, p = .006) and TMT-A (r = -.382, p = .049). In CFS without depression, impairments of cognitive performance remained with significant differences in indices of psychomotor speed (TMT-A: p = 0.027; digit-symbol substitution: p = 0.004; digit-symbol copy: p = 0.007; scanning: p = .034) Stroop test suggested differences due to processing speed rather than inhibition.

Both cohorts confirmed relationships between cognitive performance and HRV (digit-symbol copy (r = .330, p = .018), digit-symbol substitution (r = .313, p = .025), colour-naming trials Stroop task (r = .279, p = .050).

**Conclusion:**

Cognitive difficulties in CFS may not be as broad as suggested and may be restricted to slowing in basic processing speed. While depressive symptoms can be associated with impairments, co-morbidity with major depression is not itself responsible for reductions in cognitive performance. Impaired autonomic control of heart-rate associates with reductions in basic processing speed.

## Introduction

Chronic fatigue syndrome (CFS) is a chronic debilitating condition that affects at least 0.2–0.4% of the UK population and is life changing [1,2]. Despite this there are currently no diagnostic tools beyond symptom recognition and no curative treatments [2]. Almost 90% of those with CFS describe cognitive abnormalities, including poor concentration, memory loss, an inability to take in information, and a general reduction in cognitive ability [3–8]. Although these studies have identified a number of cognitive deficits, they are often of small magnitude and involve multiple domains, so no consistent profile has been identified across studies. However, a meta-analysis [9] and a recent review [10] suggest that those with CFS experience cognitive difficulties particularly in domains of attention, memory and motor speed.

Clinical heterogeneity across cohorts may explain the observed differences in the pattern and magnitude of cognitive deficits in CFS. Neuropsychiatric symptoms are often present in CFS, with the prevalence of major depression (MDD) being particularly high [11,12]. In MDD cognitive deficits have been observed in the same domains implicated in CFS, namely attention, executive function, memory and motor speed [13, 14]. Therefore an important step to accurately characterise cognitive deficits in CFS is to address this major confound. Previous evidence regarding the role of depressive symptoms in the cognitive deficits of CFS has been mixed, with some [8, 15] but not all [16, 17] studies showing a relationship.

One area that has received relatively little attention thus far is the possible role of autonomic nervous system dysfunction in the cognitive problems in CFS. Autonomic signs and symptoms are commonly [18], but not universally [19, 20], reported and may be an important component of the aetiology of CFS. Heart-rate variability (HRV) is a measure of cardiovascular autonomic control with lower HRV indicating reduced autonomic function [21]. One previous study has shown that CFS patients with lower HRV also showed poorer function in several neurocognitive tasks [16] particularly memory and attention/processing speed.

In the current study we explored potential deficits in cognitive performance in CFS. We performed studies in two separate cohorts of CFS patients. Identification and recruitment of CFS participants was identical in both phases, with all patients fulfilling Fukuda criteria [22] for CFS. In the first cohort we explored cognitive function in a heterogeneous cohort of consecutive patients attending a CFS clinical service, which allowed us to investigate the potential link with depressive symptoms. In contrast, in cohort two participants with co-morbid major depression were rigorously excluded. The purpose of these two cohorts was to allow us to establish which findings were associated with CFS and which were related to co-morbid depression. Furthermore, we investigated the relationship between cognitive performance and HRV in these cohorts of CFS in an attempt to replicate earlier findings in this area [16].

## Methods

### Cohort 1: Exploring cognitive performance in an unselected cohort of CFS patients

#### Participants

Forty eight consecutive CFS patients were recruited as part of an ME Research UK funded project via the Newcastle upon Tyne Royal Victoria Hospital clinical Chronic Fatigue Syndrome Service. All fulfilled the Fukuda diagnostic criteria [22] applied by the same Physician. Consecutive patients were provided with a Patient Information Sheet and invited to contact the research team if they were willing to be involved. Participants were not selected positively or negatively according to any criteria other than the fact that they were attending the clinical service and had a Fukuda diagnosis of CFS. All participants gave written, informed consent prior to participation and the study was approved by the Newcastle and North Tyneside NHS Research Ethics Committee.

#### Neuropsychological assessment

All participants performed of the following neuropsychological tests with a trained operator (RP):

**Intellectual Function** was measured with the Wechsler Abbreviated Scale of Intelligence (WASI) [23] to derive a full-scale intelligence quotient (IQ) as a summary of overall cognitive ability. This is a standardized assessment and established short form of the Wechsler Adult Intelligence Scale (WAIS-III). It assesses vocabulary comprehension, visuoconstructive ability (block design), verbal reasoning (similarities) and nonverbal deductive reasoning (matrices). Raw scores are compared with population age and sex normative values, resulting in a T score that is standardized to a mean of 50 and a standard deviation of 10.

**Verbal Memory** was assessed with the Logical Passages Test from the Wechsler Memory Scales III (WMS-III; [24]). This assesses immediate and delayed verbal declarative recall of a passage read once to the participant.

**Visual Memory** was assessed with the Family Pictures test from the WMS-III, which measures immediate and delayed visual memory. The test involves recalling the details from visually presented scenes both immediately and after a delay.

**Verbal Working Memory** was assessed with Digit Span (WAIS-III) [25], which requires the participant to recall verbally presented sequences of numbers in either forwards or reverse order. The task requires attention, concentration, working memory, mental control, and reasoning.

**Executive Function** was assessed with 1) Verbal Fluency [26], which requires the participant to generate as many words as possible in one minute that begin with a given letter and accord to certain rules. 2) The Trail-Making Test (TMT; [27], which is a test of complex visual scanning and which requires a combination of information processing speed, manual motor speed, and sustained attention. Part B requires participants to alternate between connecting numbers and letters, thus involving additional category switching and executive functions.

**Psychomotor Speed** was assessed with three different tests. 1) Symbol Search (WAIS-III), which requires visual scanning to identify whether target nonsense symbols appear in an array and to complete as many as possible in a given time limit. 2) Digit Symbol Coding (WAIS-III), which involves visual scanning, paired associates learning and fine-grained motor responses in order to use a key to draw nonsense symbols under their paired digit, completing as many as possible in a given time limit. 3) Part A of the TMT which requires participants to draw lines connecting consecutively numbered circles as quickly as possible.

#### Depressive symptom assessment

Depressive symptoms were assessed with the Depression subscale of the Hospital Anxiety and Depression Scale (HADS-D) [28], a clinician rated instrument to determine the level of anxiety and depression on a scale from 0 to 21 respectively. These were available for 25 of the 48 CFS participants, as this was a multi-component study and not all participants took part in each component.

#### Assessment of autonomic function using heart-rate variability

Continuous ECG recordings were taken during a supine-rest period of 10-minutes using the Task Force Monitor (CNSystems Medizintechnik AG, Graz, Austria) system in 46 of the 49 participants. Consecutive R-R intervals were recorded by the system and analysed using the RHRV toolbox [29]. The R-R interval time series was first filtered to remove intervals that were either too short or too long. A linear analysis of the filtered time series then computed the root-mean-square of successive differences (RMSSD) as a global measure of HRV and which has also been reported [16].

#### Data analysis

The performance of the CFS patients was compared with the standardised norms available for each test. Clinically, performance below the 5^th^ percentile is typically an indicator of a significant deficit. Average percentile performance and percentage of the sample performing below the 5^th^ percentile are reported. The relationship between neuropsychological performance and depressive symptoms was examined by Pearson correlations. The relationship between neuropsychological performance and HRV was examined by Person correlation with log-transformed RMSSD values, which showed a skewed distribution in their original values. Statistical analyses were conducted using SPSS version 21.

### Cohort 2: Exploring cognitive performance in CFS where depression is not a confounder

The general recruitment procedures were identical to cohort 1. Participants were recruited as part of a Medical Research Council-funded observational study aimed at understanding the pathogenesis of autonomic dysfunction in patients with chronic fatigue syndrome.

#### Participants

Patients (n = 51) were not selected positively or negatively according to any criteria other than the fact that they were attending the clinical service and had a Fukuda diagnosis of CFS. However, in this cohort patients were excluded if they screened positive for a major depressive episode as assessed by a trained Physician using the Structured Clinical Interview for the Diagnostic and Statistical Manual for Mental Disorders (version IV; SCID-IV [30]).

Controls (n = 20) were recruited via advertisement in the local hospital and University, together with a distribution of posters via the local ME Patient Support Group where the relatives of those with CFS were invited to participate. Controls were considered to be community controls rather than healthy controls, i.e. they fulfilled the same inclusion and exclusion criteria as CFS participants other than the fact that they did not have a CFS diagnosis. All participants gave written, informed consent prior to participation and the study was reviewed and approved by the Newcastle NHS ethics committee (REC 12/NE/0146, CLRN ID 97805).

#### Neuropsychological assessment

The following battery of neuropsychological assessments were performed by a trained clinician in the clinical research facility.

**Psychomotor Speed** was assessed nearly identically to study 1 using the Trail Making Test—see above for details, and the Digit Symbol Substitution Test (DSST), a modified version of the Digit Symbol Coding test [31], which alongside the standard version of the task also includes control conditions for motor speed (Digit Symbol Copy) and scanning efficiency (Digit Symbol Scanning).

**Intellectual Function** was assessed with the National Adult Reading Test (NART; [32]) which requires the participant to correctly pronounce a list of irregularly spelled words. It gives an estimate of premorbid IQ.

**Verbal Learning and Memory** was assessed with the Rey Auditory Verbal Learning Test [33], which involves recall of a word list presented multiple times and assesses both immediate recall, learning and recall after a 30minute delay.

**Verbal Working Memory** was assessed with Digit Span (WAIS-III) [25], which requires the participant to recall verbally presented sequences of numbers in either forwards or reverse order. The task requires attention, concentration, working memory, mental control, and reasoning.

**Spatial Working Memory** was assessed with the “Blocks” test of the WAMI-III which gives forward and backward spatial span measures similar to the Digit Span in the verbal domain.

**Visual Working Memory** was assessed with the Visual Patterns Test [34] which assesses short term recall of matrices of white and black squares that become increasingly complex.

**Executive Function** was assessed with the Stroop Colour-Word Test [35] which requires participants to say aloud the colour of the ink in which a colour-related word is printed (e.g. the word ‘red’ printed in green ink). This requires mental control and inhibition.

#### Heart rate variability assessment

Measurement and calculation of HRV was identical to the procedures of cohort 1, but were restricted to the patient group only.

#### Data analysis

The CFS and control groups were compared with independent samples t-tests using bias corrected and accelerated bootstrapping (with a resampling value of 1000 and the Mersenne Twister seed set to 200). The alpha level was set to 0.05. Where Levene’s test for unequal variances indicated unequal variances, the appropriate adjustment to the degrees of freedom and p-value were used. Effect sizes were calculated using Cohen’s d and were transformed such that a positive effect size indicates better performance by the control group. The relationship between neuropsychological performance and HRV in the patient group was examined by Person correlation with log-transformed RMSSD values, which showed a skewed distribution in their original values. Statistical analyses were conducted using SPSS version 21.

## Results

Phase 1: Exploring cognitive performance in an unselected cohort of CFS patients

Demographic details and scores on the neuropsychological assessment for study 1 are shown in Table 1. Characterising the *group* using age-standardised scores shows performance in the average range on most of the measures (the average percentile rank does not differ markedly from 50 and falls within 1 standard deviation of the mean for all tests; i.e. 16^th^ to 84^th^ percentile). However, after calculating the proportion of CFS patients at or below the 5^th^ percentile it was observed that between 0 and 23% of the CFS sample fall within this range across all test indices (Table 1).

**Table 1:** Phase 1 demographics and neuropsychological test performance.

	Patients (n = 48)		Patients vs norms	
	Mean	s.d.	Percentile	% below 5^th^ percentile
**Demographics**				
Age	44.9	12.3	-	-
Gender (%F)	89.6	-	-	-
HADS-D score	8.9	4.5	-	-
**Cognitive function**				
*Verbal Memory*				
Immediate—Logical Memory 1	9.6	3.4	46.8	10.4*
Delayed—Logical Memory 2	9.9	3.4	48.7	8.3
*Visual Memory*				
Immediate—Family Pictures 1	8.6	2.9	37.2	18.8**
Delayed–Family Pictures 2	8.7 ^a^	3.3	40.1	19.1*
*Intellectual Function*				
Block Design	55.7	9.2	67.4	2.1
Matrix Reasoning	57.1	8.4	71.8	2.1
Vocabulary	54.8	8.1	65.0	0
Similarities	52.8	6.8	59.1	0
Estimated Full scale IQ	109.3	12.3	68.3	0
Symbol Search	10.2 ^a^	2.2	52.2	0
Digit Symbol Coding	9.1	2.8	41.3	10.4*
Digit Span	11.3	2.9	60.1	0
*Psychomotor Speed*				
Trail Making Test A	35.7^a^	14.9	38.9	21**
*Executive Function*				
Trail Making Test B	69.9 ^a^	25.8	41.9	23**
Verbal Fluency	43.1	12.5	63.0	2.1

^a^ n = 47 due to missing data

*above expected ratio (p < .1)

**above expected ratio (p < .01)

### Relationship with depressive symptoms

Of the 25 patients who completed a battery of self-report questionnaires as part of this multi-component study, 11 patients met the HADS cut-off score for depression. There were no significant differences in age (t_57_ = 1.49, p = 0.141), gender (χ^2^_1_ = 0.037, p = 0.848) or neuropsychological test performance for those who completed the HADS compared to those who did not (all t<1.90, all p>0.05). Similarly there were no significant differences in age (t_23_ = -0.95, p = 0.352) and gender (χ^2^_1_ = 0.032, p = 0.859) between those that scored above and below the cut-off. Scores for tests that showed a proportionally higher than expected percentage of patients falling below the 5^th^ percentile of the age-standardized score were correlated with HADS-D scores. Significant negative correlations were found with Digit-Symbol-Coding (r = -.507, p = .006) and TMT-A (r = -.382, p = .049).

### Relationship with HRV

Log-transformed RMSSD values correlated positively with both the raw (r = .425, p = .004) and scaled (r = .311, p = .038) scores of the WAIS-III symbol search task, and positive with the raw score of the WAIS-III symbol substitution task (r = .347, p = .018) but not the scaled score (r = .224, p = .135). It correlated negatively with time taken on the TMT-B (r = -.309, p = .039).

Phase 2: Exploring cognitive performance in CFS where depression is not a confounder

Demographic details and scores on the neuropsychological assessment are shown in Table 2. There were significant differences between the groups in several indices of psychomotor speed (Trail making test part A, time to complete: Cohen’s d = 0.65, p = 0.027; digit-symbol substitution, symbols per minute: d = 0.78, p = 0.004; digit-symbol copy: d = 0.80, p = 0.007; digit-symbol scanning: d = 0.67, p = .034); verbal learning (delayed verbal recall: d = 0.49, p = 0.041); and executive function (Stroop colour-word trial number correct: d = 0.74, p = 0.002). Comparing performance of the groups on the two control indices of the Stroop test (word reading and colour naming) showed significant differences, which suggests differences on the colour-word trial were likely due to speed of processing rather than difficulties with inhibition.

**Table 2:** Phase 2 demographics and neuropsychological test performance.

	Patient		Control				
	Mean	s.d.	Mean	s.d.	Mean difference (95%CI)	p	d
*Demographics*							
Age	46.0	11.9	44.8	16.5	-1.11 (-9.19,6.80)	0.785	
Gender (%F)	75%	-	65%	-	χ^2^ = 0.642	0.423	-
*Premorbid IQ*							
NART	117.9	7.9	117.5	9.2	-0.41 (-4.79,3.93)	0.851	-0.05
*Verbal Memory*							
Digit Span forwards	6.8	1.4	6.6	1.5	-0.16 (-0.59,0.92)	0.676	-0.12
AVLT total recall (trails 1–5)	46.9	10.4	49.4	7.9	2.46 (-1.78,6.1)	0.281	0.25
AVLT recall after interference (A6)	9.6	3.3	10.7	2.4	1.06 (-0.30,2.36)	0.125	0.34
AVLT delayed recall (A7)	9.1	3.2	10.7	2.7	**1.51 (0.06,3.01)**	0.041*	0.49
AVLT recognition (list A hits)	12.4	2.5	12.9	2.3	0.44 (-0.86,1.65)	0.477	0.18
*Visual Memory*							
Visual Patterns Test total score	22.5	5.9	23.5	6.4	1.03 (-2.17,4.20)	0.559	0.17
*Spatial Memory*							
Spatial Span forwards	5.1	1.5	5.6	1.7	0.50 (-1.35,0.36)	0.229	0.33
Spatial Span backwards	4.9	1.2	5.0	1.2	0.08 (-0.61,0.71)	0.828	0.06
*Executive Function*							
Verbal Fluency (total correct)	39.3	10.6	44.8	12.2	5.50 (-0.19,11.06)	0.075	0.50
Stroop Colour-Word trial (total correct)	37.9	12.4	46.9	11.8	**9.02 (2.44,15.35)**	0.006*	0.74
Stroop Interference score	2.5	8.3	2.8	9.1	-0.24 (-5.04,4.92)	0.926	-0.03
Digit Span backwards	5.0	1.3	5.4	1.2	0.42 (-0.23,0.99)	0.186	0.34
Trail Making Test B	63.9	37.1	55.2	20.1	-9.15 (-21.69,2.97)	0.236	0.31
Trail Making Test B-A	32.9	23.1	33.1	19.2	0.17 (-9.29,11.16)	0.977	-0.01
*Psychomotor Speed*							
Trail Making Test A	30.6	17.4	22.2	6.4	**-8.47 (-15.00,-3.30)**	0.027*	0.65
Digit-Symbol Substitution (1/min)	35.2	9.5	42.7	9.8	**7.52 (2.78,13.25)**	0.004*	0.78
Digit-Symbol Copy (1/min)	67.5	18.9	82.7	19.7	**15.24 (5.22,24.70)**	0.007*	0.80
Digit-Symbol Scanning (1/min)	37.9	8.6	45.5	13.6	**7.55 (1.69,14.24)**	0.034*	0.67
Stroop Colour Reading (total correct)	84.2	25.0	104.3	14.6	**20.06 (11.07, 29.09)**	0.001*	0.98
Stroop Colour Naming (total correct)	61.6	17.0	76.4	11.3	**14.71 (8.01,21.52)**	0.001*	1.02

*Statistically significant result

### Relationship with HRV

Log-transformed RMSSD values correlated positively with digit-symbol copy (r = .330, p = .018) and digit-symbol substitution (r = .313, p = .025). It further correlated positively with performance on the colour-naming trials of the Stroop task (r = .279, p = .050). No other correlation of RMSSD with neuropsychological measures reached statistical significance.

## Discussion

Our study suggests consistent cognitive impairments in CFS which is shown in both cohorts, including cohort 2 in which patients with depression had been rigorously excluded.

That cognitive impairment correlated with the depression scale score in cohort 1 but was still present in cohort 2 is interesting and perhaps suggests that depression explains some but not all of the cognitive impairment in CFS. Cohort 1 used the HADS scale which is commonly used in CFS populations [36–38], however, its ability to discriminate between general disease burden and core affective symptoms has not been established; this is exemplified by the question “I feel as if I am slowed down”-. It is a limitation of the study that sub-threshold depressive symptoms were not recorded in cohort 2. In a previous study, correlations between depression and attention and between depression and executive function disappeared once overall fatigue severity was statistically accounted for; however, a correlation between depression and verbal memory remained [8].

Taken together, our two cohorts suggest an impairment in psychomotor speed, with reduced performance in various measures of memory. Cohort 1 showed three memory indices with an elevated proportion of patients with clinically significant reductions in performance. In cohort 2 only one memory measure (delayed free recall) showed significantly weaker performance in the patient group compared to the control group. This suggests that memory deficits in CFS are less consistent and may perhaps depend on the exact nature of the tasks used to assess memory function.

The consistent evidence in these two cohorts of reduced psychomotor speed suggest a general slowing of either basic perceptual-cognitive processes, basic motor responses or both. The finding that the largest effect sizes are seen in sub-tests that should make minimal cognitive demands, such as the symbol copy of the digit-symbol task and the two control conditions of the Stroop task, support the hypothesis that the core neuropsychological deficits in CFS may mostly be related to slow processing speed. Such slowing could potentially account for deficits in other tasks that require speeded responses. A meta-analysis and a recent systematic review have shown that general slowing of processing speed in attention tasks in CFS patients is a consistent finding and shows the largest effect size when comparing CFS patients to healthy volunteers [9, 10]. It is further clear from our findings that such slowing cannot be explained by potential co-morbid major depression although depressive symptoms may exacerbate the processing speed deficit.

A further finding that is consistent with a previously published study [16] is the association between a measure of resting HRV and indicators of psychomotor speed in the variants of the digit-symbol task. While additional associations of cognitive tasks with HRV have been found in our two cohorts and in the previous study, only the digit-symbol task has consistently produced significant correlations with RMSSD in all three samples. Given the correlative nature of these findings and that we did not apply error correction to these analyses one of course has to be cautious when interpreting them. The findings do suggest however that CFS patients with larger processing-speed deficits tend to also be patients who exhibit reduced autonomic control of cardiovascular function. How these two deficits are linked remains unknown. One possible scenario is that both are caused by a common underlying disease process. Beaumont and colleagues [16], for instance, proposed chronic loss of inhibitory control by the prefrontal cortex as a possible cause for both the reduced autonomic function and the impaired cognitive function, and characterized CFS as a potential “state of physiological hyper-vigilance”. However, whereas Beaumont and colleagues showed significant associations between HRV and all of their neurocognitive tasks (digit symbol, spatial working memory, Stroop) [16], in our two cohorts we only found consistent associations with the digit symbol task, and only isolated and inconsistent associations to the other tasks. Thus, it is not clear how the proposed model would explain an apparent specificity to measures of processing speed. Neuroinflammation is an alternative common pathophysiological process for both the ANS dysregulation and the cognitive impairment. Cognitive difficulties in CFS have recently been associated with brain measures of neuroinflammation [39] particularly in sub-cortical brain structures such as the thalamus and amygdala. Both of these structures have been implicated in basic cognitive function, particularly attention, and central autonomic control. However, as the above study used self-reported measures of cognitive (dys)function it is unclear how these might relate to objective measures of cognitive performance, as there is no clear relationship between subjectively reported cognitive problems and objective, laboratory based measures of cognitive function in CFS [9].

The fact that the current studies found only limited evidence for broad neurocognitive impairment in CFS may seem surprising given the suggestion that neurocognitive dysfunction is one of three core symptoms that are present in about 95% of CFS patients [40]. The above mentioned lack of relationship between subjectively reported and objectively measured cognitive difficulties may account for this apparent discrepancy [9]. Laboratory based measures of cognition may not fully capture the degree with which patients are (or perceive themselves to be) cognitively impaired in everyday life. Methodological differences have also been hypothesized as a potential source for discrepancies in findings between studies [10]. Tasks that require speeded responses may be more sensitive to an underlying (core) deficit in processing speed, and may therefore be more likely to show differences between CFS patients and controls.

This study has a number of limitations. Measures of symptom severity and education levels may impact upon cognitive performance and should be considered in future research. Cohort 1 and 2 described depressive symptomatology using different tools and although the staged approach to our protocol allowed associations to be considered it does not allow direct testing of a hypothesis. CFS remains a poorly defined heterogenous condition with subjective diagnostic criteria, research to characterise CFS phenotypes is needed.

## Conclusion

This study showed that cognitive difficulties in CFS may not be as broad as sometimes suggested and may instead be restricted to a slowing in basic processing speed, with potential follow-on effects on other cognitive domains (depending on specific test parameters). While depressive symptoms can be associated with these impairments, co-morbidity with major depression is not itself responsible for these reductions in cognitive performance. In addition, the present study provided further evidence that impaired autonomic control of heart-rate is associated with reductions in basic processing speed, although how exactly these two findings are linked remains unknown.

## Supporting information

S1 Dataset Data is included as S1_Dataset. (XLSX)

## Abbreviations

CFS: Chronic fatigue syndrome
MDD: Major depression
SCID: Structured Clinical Interview for Depression
HRV: Heart rate variability
TMT: Trail Making Test
HAD: Hospital Anxiety and Depression
